# Optical Coherence Tomography based indices in predicting functional outcome of macular hole surgery: A retrospective chart review

**DOI:** 10.12669/pjms.37.5.4126

**Published:** 2021

**Authors:** Haroon Tayyab, Rehman Siddiqui, Sana Jahangir, Shiraz Hashmi

**Affiliations:** 1 Dr. Haroon Tayyab, FCPS (Ophth), FCPS (VRO), FRCS (Glasg), FRCSEd Section of Ophthalmology, Department of Surgery, The Aga Khan University, Karachi, Pakistan; 2 Dr. Rehman Siddiqui, MRCOphth, FRCOphth Section of Ophthalmology, Department of Surgery, The Aga Khan University, Karachi, Pakistan; 3 Dr. Sana Jahangir, MD, FCPS Department of Ophthalmology, Allama Iqbal Medical College, Jinnah Hospital, Lahore; 4 Dr. Shiraz Hashmi, MBBS, M.Sc Section of Ophthalmology, Department of Surgery, The Aga Khan University, Karachi, Pakistan

**Keywords:** full thickness macular hole, vitrectomy, optical coherence tomography, external limiting membrane, macular hole index

## Abstract

**Objectives:**

The objective of this study was to assess the utility of novel macular hole indices of Optical Coherence Tomography (OCT) and predicting the functional outcome of surgery.

**Methods:**

This was a retrospective chart review of 28 eyes who underwent surgery for idiopathic Full Thickness Macular Hole (FTMH) at The Aga Khan University Hospital (AKUH), Karachi from January 2016 to March 2020. Data of preoperative OCTs were recovered from data server of OCT machine. Measurements of the pre-operative OCTs were calculated using caliper function of OCT software by two independent technicians. Parameters included Macular Hole Index (MHI), Traction Hole Index (THI), Hole Form Factor (HFF) and Diameter Hole Index (DHI) were recorded. Receiver operating characteristic (ROC) curve was used to evaluate the performance of DHI, THI, HFF and MHI for improved BCVA after surgery, by looking at sensitivity, specificity and area under curve (AUC). P-value of <0.05 was considered significant.

**Results:**

Out of 30 eyes, final data analysis was done for 28 eyes. Mean age was 61.5 ± 6.2 years. Mean pre-operative and 6 months post-operative LogMAR best corrected visual acuity (BCVA) was 0.84 ± 0.23 and 0.32 ± 0.30 (p-value <0.001). Area under the curve with 95% confidence interval estimated for DHI, THI, HFF, and MHI was [0.750 (0.559 to 0.889)], [0.827 (0.637 to 0.943)], [0.846 (0.660 to 0.954)], [0.827 (0.637 to 0.943)]. Cut off values for predicting good functional outcome (post-op BCVA equal or better that 0.4) for DHI, THI, HFF and MHI were 0.454, 1.086, 0.856 and 0.501 respectively. All ROC value of less than 0.5 were considered unlikely to predict functional outcomes with macular hole indices.

**Conclusion:**

Novel macular hole indices can be used as a tool to predict the functional outcomes of macular hole surgery. Larger studies may be required to assess their wider effectiveness.

## INTRODUCTION

Full thickness macular Hole (FTMH) is a relatively common macular pathology with an incidence of 7.8 per 100,000 per annum.^1^ Usually, it occurs in middle to old age groups and is not accompanied with any other retinal pathology.^2^ Female to male ratio is 2:1 in reported literature.^3^

The standard of care for FTMH has been pars plana vitrectomy (PPV) with internal limiting membrane (ILM) peeling and intra ocular gas injection for last many year.^4^ Visual and anatomical outcome for FTMH surgery has been known to depend on the size of the hole, initial best corrected visual acuity (BCVA) and duration of macular hole formation 5. Recently, optical coherence tomography (OCT) based indicators have been used and assessed for the ability to predict both functional and structural outcomes after macular hole surgery.^6^ This has advanced the role of OCT from just diagnosing FTMH towards predicting outcome. This application of different FTMH parameters based on pre-operative OCT can give us further insight into the pathogenesis of FTMH and effectiveness of varying surgical techniques in yielding better surgical outcomes.

Researchers have used Diameter Hole Index (DHI), Tractional Hole Index (THI), Hole Form Factor (HFF), minimum hole diameter, hole base diameter, maximum hole diameter, apex diameter, macular hole height, hole arm length and Macular Hole Index (MHI) as OCT based indicators to predict structural and functional outcome of FTMH surgery.^7^-^9^ Similarly, other OCT based findings may correlate with the functional outcome of macular hole surgery like the integrity of External Limiting Membrane (ELM) and Inner Segment/Outer Segment (IS/OS) line.^10^

The pioneering work in evaluation of OCT based indicators for FTMH prognosis were first published by Ip et al and since then, there have been some reports on different indices that have been studied for the same purpose.^11^-^13^ These indices incorporate the traction forces on macular hole in predicting surgical outcomes. For example, THI is a ratio between maximum height to minimum hole diameter and accounts for antero-posterior traction where DHI signifies tangential traction on hole by evaluating the ratio of minimum hole diameter and base diameter 14. Other indices like MHI and HFF can take into account both tangential and antero-posterior tractional forces in their calculations. Tractional Hole index has been positively co related with better visual outcomes at 3 months after macular hole surgery.^14^

To the best of our knowledge, these indices have not been studied in Pakistani population. We aim to study these OCT based parameters in our population in predicting the functional outcome of FTMH surgery and compare the results with international literature to explore the utility of this information in our population of FTMH. We also plan to analyze the value of these parameters against previously well understood parameters used for prognostic purpose.

## METHODS

This study was designed as retrospective chart review of 30 eyes diagnosed to have FTMH and then proceeded with surgical management. The cases reviewed in this study were from January 2016 to March 2020. All of these cases presented at The Aga Khan University Hospital, Karachi, Pakistan.

These cases were diagnosed clinically on slit lamp examination by an experienced vitreoretinal surgeon. Further macular hole staging and parameter measurement was done using Spectral Domain Optical Coherence Tomography (SD-OCT – Spectralis^®^ Heidelberg Engineering Inc. Franklin, USA). This parameter measurement was done using caliber function of OCT machine in all pre-operative OCT scans by two independent operators and then verified by an experienced vitreoretinal surgeon. Patients who had other concurrent diseases that may impair the functional outcome of surgery were excluded from the study (glaucoma, retinal detachment, vascular retinopathy, proliferative vitreoretinopathy, high myopia > -6.00 DS). Secondary macular holes were also excluded from the study (trauma, high myopia, secondary epiretinal membrane associated macular hole). Patients with a history of symptoms of more than six months were not included in this study. The Ethical Review Committee (ERC) of The Aga Khan University Hospital gave approval of this study. The reference number of ERC is 2020-5158-11463.

### Surgical Steps

A standard 3 port 25-gauge pars plana vitrectomy was performed by a single retinal surgeon using Constellation^®^ Vision System (Alcon Inc. Fort Worth, USA). The basic surgical steps included core vitrectomy, staining internal limiting membrane (ILM) with Brilliant Blue G dye 0.025% (ILM-Blue^®^ D.O.R.C International, ZG, The Netherlands), ILM peeling of 3-4 mm around macular hole and tamponade with 16% C3F8 gas (GOT Multi C3F8. ALCHIMIA SRL. Viale Austria). Face down positioning for 50 minutes for every one hour for seven days was advised. All patients who had cataracts also underwent phacoemulsification with intraocular lens (IOL) implant (MA60AC. Alcon Inc. Fort Worth, USA). Although patients were examined at standard time intervals as routine follow ups after surgery, the final best corrected visual acuity (BCVA) for analysis purpose was recorded at post-operative 6 month’s time. At the time of data analysis, the BCVA was converted from Snellen score to decimal LogMAR value for ease of analysis.

**Fig.1:**
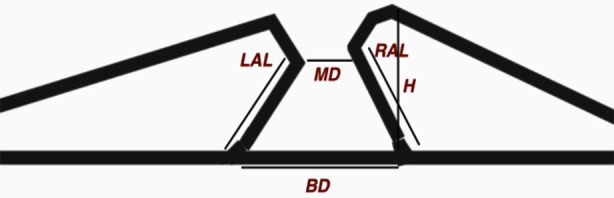
Schematics of macular hole dimensions as seen on OCT.

The OCT based parameters were measured in the following manner as shown in Fig.1.

- MD = Minimum Diameter
- BD = Base Diameter
- BH = Height
- LAL = Left Arm Length
- RAL = Right Arm Length

### Various OCT based ratios were calculated using following formulas:

- Diameter Hole Index (DHI) = MD/BD
- Tractional Hole Index (THI) = H/MD
- Hole Form Factor (HFF) = (RAL + LAL)/BD
- Macular Hole Index (MHI) = H/BD

### Statistical Analysis

The statistical analysis was performed using SPSS 20.0 (IBM software company, Armonk, NY) software. Parameters such as gender and age were described as percentages and mean with standard deviations. Pre-operative and six months post-operative BCVA was recorded in LogMAR and results were analyzed using Wilcoxon-rank sum methods. Also, receiver operating characteristic (ROC) curve was used to evaluate the performance of DHI, THI, HFF and MHI for improved BCVA after surgery, by means of specificity, sensitivity and area under curve (AUC). Only AUC more than 0.5 was taken as having effective prognostic value. P-value of <0.05 taken as statistically significant.

All data of patients was retrieved using Hospital Information System (HIM) and values were recorded using pre decided study proforma.

## RESULTS

We analyzed the data of 28 out of 30 patients. The mean age was 61.5 ± 6.2 years. The female to male ratio was 2.11:1. Mean Minimum Diameter (MD) was 448.3 ± 189.9 μm. Mean hole height was 456.2 ± 112.6 μm. Hole base diameter was 888.9 ± 277.1 μm. Table-I shows pre-operative and six months post-operative BCVA in LogMAR with a p-value <0.001 which is statistically significant.

**Table-I:** Pre-operative and post-operative BCVA.

Outcome measures	Pre-Op Mean ± SD)	6 months Post-Op Mean ± SD)	Difference (Mean ± SD) (p value)
Visual acuity (logarithm)	0.84 ± 0.23	0.32 ± 0.30	0.53 ± 0.31, p=<0.001

**Fig.2:**
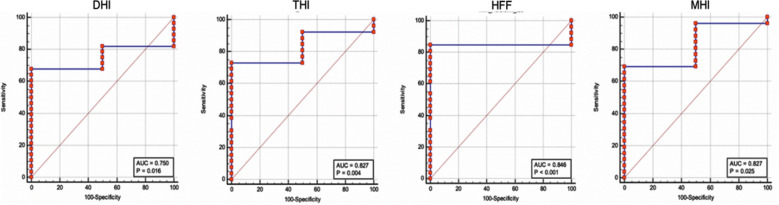
AUC for CHI, THI, HFF and MHI.

Indices of various OCT cut points derived from ROC curve analysis that predict favorable outcomes (BCVA equal to or better than 0.4 logMAR at 6 months post-operative) was: 0.454, 1.086, 0.854, and 0.501 for Diameter hole index (DHI), Tractional hole index (THI), Hole form factor (HFF) and Macular hole index (MHI) respectively as shown in Fig.2.

Area under the curve and 95% estimated to be acceptable range for DHI [0.750 (0.559 to 0.889)], however was in excellent range [0.827 (0.637 to 0.943)], [0.846 (0.660 to 0.954)], [0.827 (0.637 to 0.943)] for THI, HFF, and MHI respectively. Specificity was 100% for each indices and sensitivity was high for HFF (84.6%) and lowest for DHI (64.29%) as shown in Table-II.

**Table-II:** Cut Point for various indices with AUC, sensitivity and specificity.

Parameter	Criterion (cut point)	Area under the cure with 95% CI	Sensitivity (%) with 95% CI	Specificity (%) with 95% CI	Positive Predictive value (%)	Negative predictive value (%) with 95% CI
Diameter hole index (THI) n=28	0.454	0.750 (0.559 to 0.889)	64.29 (44.1 - 81.4)	100.00 (15.8 - 100.0)	100.0	16.7(10.8 - 24.7)
tractional hole index (DHI) n=28**	1.086	0.827 (0.637 to 0.943)	73.08 (52.2 - 88.4)	100.00 (15.8 - 100.0)	100.0	22.2 (13.2 - 35.0)
Hole form factor n=28**	0.854	0.846 (0.660 to 0.954)	84.62 (65.1 - 95.6)	100.00 (15.8 - 100.0)	100.0	33.3 (16.9 - 55.2)
Macular hole index (MHI) n=28**	0.501	0.827 (0.637 to 0.943)	69.23 (48.2 - 85.7)	100.00 (15.8 - 100.0)	100.0	20.0 (12.3 - 30.8)

## DISCUSSION

Various OCT based pre-operative parameters have been extensively studied previously in predicting the functional and structural outcome of macular hole surgery. Pre-operative minimum hole diameter and base hole diameter have been identified as negatively correlated with successful outcomes.^9^,^11^ There have been studies where different ratios of preoperative macular hole OCT have been employed to predict the outcomes of the surgery. We have also employed these OCT based parameters in predicting the functional outcomes of macular hole surgery.

The cut off values for MHI, DHI, HFF and THI in our study were 0.501, 0.454, 0.854 and 1.086 respectively. These cut off values showed a predicted LogMAR vision gain of 0.4 or better at six months post-operative time. These cut offs were associated with area under curve (AUC with 95% confidence interval), sensitivity (with 95% confidence interval) and specificity (with 95% confidence interval) of 0.827, 69, 100 for MHI; 0.75, 64, 100 for THI; 0.846, 84, 100 for HFF; 0.827, 73 , 100 for DHI. This result is in comparison with Geng et al where cut off values for MHI and HFF were 0.427 and 1.02 with comparable sensitivities and specificities. The difference was the BCVA at 6 months. We restricted the outcome to 0.4 LogMAR or better as success where Geng included all cases as success where post-operative BCVA improved by 2 lines at 6 months post-operative period.^15^ In another study by Dai et al, the cut offs for MHI and THI were 0.47 and 0.97 which correlated with improved functional results after surgery. These results are also comparable with our study as the author set the cut off for BCVA at 0.25 whereas in our study, it was 0.4.^16^

In another study, MHI of >0.5 was taken as a significant indicator for better macular hole surgical outcomes. These results are also comparable to this study.^17^ In another study, MHI and THI were considered as reliable indicators in predicting better outcomes of macular hole surgery. They noted higher values of AUC for MHI and THI (0.791 and 0.840 respectively) which is comparable to the results of our study. It was noted that MHI which incorporates the height of the hole as better predictor of improved BCVA in post-operative time as compared to HFF that takes into account the hole arm length measurements.^14^

### Limitation of the Study

The limitations of this study were its retrospective design, small sample size and relatively short follow up period. But this study was conducted in a single center with a single surgeon and utilized standardized operative techniques and post-operative rehabilitation. Newer literature is measuring additional OCT based parameters that include various volume analysis which could not be done in this study due to software limitations.

## CONCLUSION

THI and MHI are useful indicators in predicting the post-operative functional outcomes of macular hole surgery. Whether these indicators can be applied to other etiologies of macular hole (myopia, trauma) needs to be explored with larger sample size.

### Authors Contribution:

**HT:** Conceived the idea of the study and prepared the manuscript

**RS:** Performed literature search and review of manuscript

**SJ:** Performed the literature search and bibliography

**SH:** Did data analysis
